# Evaluating the Phylogenetic Status of the Extinct Japanese Otter on the Basis of Mitochondrial Genome Analysis

**DOI:** 10.1371/journal.pone.0149341

**Published:** 2016-03-03

**Authors:** Daisuke Waku, Takahiro Segawa, Takahiro Yonezawa, Ayumi Akiyoshi, Taichiro Ishige, Miya Ueda, Hiroshi Ogawa, Hiroshi Sasaki, Motokazu Ando, Naoki Kohno, Takeshi Sasaki

**Affiliations:** 1 Graduate School of Human and Animal-Plant Relationships, Tokyo University of Agriculture, Funako, Atsugi, Kanagawa, Japan; 2 National Institute of Polar Research, Midori-cho, Tachikawa-shi, Tokyo, Japan; 3 Transdisciplinary Research Integration Center, Toranomon, Minato-ku, Tokyo, Japan; 4 School of Life Sciences, Fudan University, SongHu Rd., Shanghai, China; 5 School of Advanced Science, The Graduate University for Advanced Studies, Shonan, Hayama-cho, Miura-gun, Kanagawa, Japan; 6 NODAI Genome Research Center, Nodai Research Institute, Tokyo University of Agriculture, Sakuragaoka, Setagaya-ku, Tokyo, Japan; 7 Yokohama Zoological Gardens, Kamishirane-cho, Asahi-ku, Yokohama-shi, Kanagawa, Japan; 8 Department of Contemporary Social Studies, Chikushi Jogakuen University, Ishizaka, Dazaifu, Fukuoka, Japan; 9 Department of Geology and Paleontology, National Museum of Nature and Science, Tokyo, Amakubo, Tsukuba, Ibaraki, Japan; 10 Graduate School of Life and Environmental Sciences, University of Tsukuba, Tennoudai, Tsukuba, Ibaraki, Japan; BiK-F Biodiversity and Climate Research Center, GERMANY

## Abstract

The Japanese otter lived throughout four main Japanese islands, but it has not been observed in the wild since 1979 and was declared extinct in 2012. Although recent taxonomic and molecular phylogenetic studies suggest that it should be treated as an independent species, International Union for Conservation of Nature Red List considers it as subspecies of *Lutra lutra*. Therefore, the taxonomic status of this species needs to be resolved. Here we determined the complete mitochondrial genome of two Japanese otters caught in Kanagawa and Kochi prefectures and five Eurasian otters (*L*. *lutra*). We reconstructed a molecular phylogenetic tree to estimate the phylogenetic position of the Japanese otter in Lutrinae using the Japanese otters and the other 11 Lutrinae species on the basis of *ND5* (692 bp) and cytochrome *b* (1,140 bp) sequences. We observed that the two Japanese otters had close relationships with Eurasian otters, forming a monophyletic group (100% bootstrap probability). To elucidate detailed phylogenetic relationships among the Japanese and Eurasian otters, we reconstructed a maximum likelihood tree according to mitochondrial genome sequences (14,740 bp). The Japanese otter (JO1) collected in Kanagawa was deeply nested in the Eurasian otter clade, whereas the Japanese otter (JO2) collected in Kochi formed a distinct independent lineage in the *Lutra* clade. The estimated molecular divergences time for the ancestral lineages of the Japanese otters was 0.10 Ma (95%: 0.06–0.16 Ma) and 1.27 Ma (95%: 0.98–1.59 Ma) for JO1 and JO2 lineages, respectively. Thus, JO1 was identified as a member of *L*. *lutra*; JO2 represented the old Japanese otter lineage, which may be a distinct new species or subspecies of *Lutra*. We suggest that the ancestral population of the JO2 lineage migrated to Japan via the land bridge that existed between western Japanese islands and Asian continent at 1.27 Ma.

## Introduction

Lutrinae (Mammalia, Carnivora) includes 13 species of otters, which are distributed in four continents (South America, North America, Africa, and Eurasia) and their surrounding islands [1,2]. In the species of Lutrinae, four Eurasian species, namely *L*. *lutra* (Eurasian otter), *Lutra sumatrana* (hairy-nosed otter), *Lutrogale perspicillata* (smooth-coated otter), and *Aonyx cinerea* (oriental small-clawed otter) [3], are listed as Endangered–Near Threatened in the International Union for Conservation of Nature (IUCN) Red List (http://www.iucnredlist.org/). The IUCN Red List recognized that the following seven subspecies exist in the species of *L*. *lutra*; 1) *L*. *l*. *lutra* living in Europe and northern Africa: 2) *L*. *l*. *nair* living in southern India and Sri Lanka: 3) *L*. *l*. *monticola* living in northern India, Nepal, Bhutan, and Myanmar: 4) *L*. *l*. *kutab* living in northern India: 5) *L*. *l*. *aurobrunnea* living in northern India and Nepal: 6) *L*. *l*. *barang* living in southeast Asia: and 7) *L*. *l*. *chinensis* living in southern China and Taiwan.

**Fig 1:**
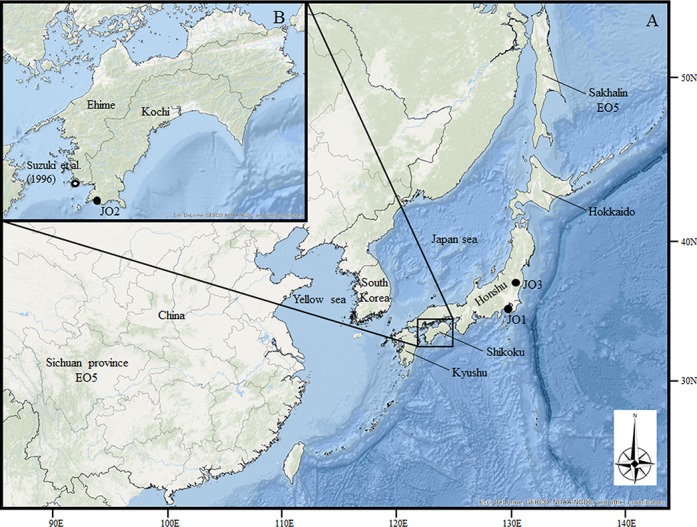
Map showing the locations of Japanese and Eurasian otters. (A) Map of East Asia. (B) Map of Shikoku Island. The capture locations of the individual Japanese otters used in this study are indicated by filled circles (JO1, JO2, and JO3). The capture location of the individual Japanese otter used by Suzuki et al. [10] is indicated by an open circle. EO indicates the locations from where Eurasian otter samples were obtained (EO3, EO5). Reprinted from PLOS ONE under a CC BY license, with permission from Environmental Systems Research Institute, Inc. (Esri), original copyright 2015.

Until the 1920s, otters were also distributed widely throughout the four main Japanese islands (Hokkaido, Honshu, Shikoku, and Kyushu, Fig 1) [4]. However, there have been no reported sightings of this animal in the wild since 1979 [5,6]. In 2012, the Ministry of the Environment of Japan announced that the Japanese otter was extinct in Japan. Taxonomically, the Japanese otter was initially classified as a subspecies of *L*. *lutra* and named *Lutra lutra whiteleyi* [7]. Later, Imaizumi and Yoshiyuki [8] re-examined its taxonomic status on the basis of morphometric analysis using 15 Japanese otter skulls (seven specimens from Honshu, six specimens from Shikoku, and two specimens from Hokkaido) and proposed that the Japanese otters from Honshu and Shikoku Islands should be classified as a distinct species *Lutra nippon*, whereas the Japanese otters from Hokkaido Island should be classified as a subspecies of the Eurasian otter *L*. *l*. *whiteleyi*. Endo et al. [9] additionally re-examined the taxonomic status of the Japanese otter on the basis of osteometric analysis using seven skulls of otters that were caught in Shikoku. In their analysis, five of the skulls were newly analyzed, and they suggested that the morphological characteristics of the Japanese otter clearly differed from that of the Chinese populations of *L*. *lutra* and *L*. *l*. *chinensis* living in Taiwan. However, their studies did not analyze the Japanese otter from Honshu and six subspecies of *L*. *lutra*.

On the other hand, Suzuki et al. [10] analyzed the molecular phylogenetic status of a Japanese otter from Shikoku Island (Fig 1B) on the basis of comparisons with one individual of *L*. *l*. *lutra* from Latvia, one individual of *L*. *l*. *chinensis* from Sichuan province, China, two individuals of *L*. *lutra* (unknown subspecies), and one *A*. *cinerea* specimen using the partial mitochondrial DNA (mtDNA) cytochrome *b* (*cytb*) sequences (224 bp), which suggested that the Japanese otter formed a distinct and independent lineage as a sister group to a monophyletic group of the other four specimens of *L*. *lutra*. Their study demonstrated that the Japanese otter should be treated as an independent species, as suggested by Imaizumi and Yoshiyuki [8]. However, the sequence length that they analyzed was quite short; using a large amount of sequence data is effective to get closer to the true phylogeny [e.g. mitochondrial genome (mtGenome) sequence] [11]. Furthermore, in the process of sequence determination for the Japanese otter, they obtained two types of *cytb* and one *cytb*-like clones, using subcloning of the polymerase chain reaction (PCR) product. However, they could not identify an orthologous sequence of the *cytb* gene from those candidates. It is necessary to verify an orthologous sequence of *cytb* gene for estimating phylogenetic relationships among the Japanese otter and its related species. Koepfli et al. [12] investigated the phylogenetic relationships among 11 species of Lutrinae on the basis of the *cytb* and NADH dehydrogenase subunit 5 (*ND5*) genes, where they used 41 *L*. *lutra* individuals from Europe and Korea. However, their analysis did not include specimens from the Japanese otter. Despite the claims that the Japanese otter should be treated as an independent species, the IUCN Red List treats *L*. *nippon* as synonym of *L*. *lutra* pending further review; therefore, the taxonomic status of the Japanese otter remains controversial.

Recently, ancient DNA studies from extinct animal specimens, e.g., the Elephant bird and the Tasmanian tiger have been carried out by high-throughput, “next-generation” DNA sequencing (NGS) technologies. These studies provided insight into the evolutionary history of those extinct species by constructing a molecular phylogenetic tree [13,14].

In this study, we analyzed museum specimens of the extinct Japanese otter from Honshu and Shikoku, and determined mtGenomes of those specimens using NGS technology. We characterized mtGenome sequence of the Japanese otter in comparison with those of Lutrinae species. Furthermore, to clarify taxonomic status of the Japanese otter, we estimated phylogenetic relationships among the Japanese otter and its related species on the basis of a partial or complete mtGenome sequence. We also calculated molecular divergence time of the Japanese otter and inferred their evolutionary history.

## Materials and Methods

### Ethics Statement

Eurasian otter is listed as Near Threatened in the IUCN Red List, and Appendix I of the Convention on International Trade in Endangered Species of Wild Fauna and Flora. Four tissue samples were provided by Noichi Zoological Park (NZP) in Kochi Prefecture, Toyama Municipal Family Park Zoo, Yokohama Zoological Gardens, and National Museum of Nature and Science, Tokyo. The animals died of natural causes in the three in Japanese zoos; Sakhalin sample was a carcass found in wild, and was imported to the National Museum of Nature and Science, Tokyo before Japan and Russia ratified the Convention on International Trade in Endangered Species of Wild Fauna and Flora. Japanese otter is an extinct species in Japan. Tissue samples were provided by NZP, Yokosuka City Museum, and Agriculture and Forestry Research Institute, Shizuoka Prefecture. Permission was obtained from all zoos, museums, and institutions to access the specimens and all samples were on loan for scientific purposes. Hence, we did not kill any animals for this study.

### Extant Species Samples

Tissue samples from Eurasian otters were collected from five individuals in three zoos and one museum (Table 1) as follows. (1) EO1 and EO4 were donated by NZP, Japan. EO1 was a male of unknown origin. EO4 was a female and its mother came from China. (2) EO2 was donated by Yokohama Zoological Garden, Japan. EO2 was a male individual and its mother came from China. (3) EO3 was *L*. *l*. *chinensis* that was donated by Toyama Municipal Family Park Zoo, Japan. EO3 was a male individual and its mother came from Sichuan province, China. (4) EO5 was donated by the National Museum of Nature and Science, Tokyo, Japan. The sex of EO5 was unknown, but it came from Sakhalin, Russia. Taxonomic status of subspecies of the EO1, EO2, EO4, and EO5 were unknown. Total genomic DNA was extracted from these Eurasian otters using standard phenol–chloroform methods [15].

**Table 1:** Origins of the Eurasian and Japanese otters used in this study.

	Sample	Subspecies	Studbook number or voucher	Sex	Locality	Sample type	Seq. approach	Accession No.
**Eurasian otter(*Lutra lutra*)**	**EO1**	**unknown**	**#32**	**Male**	**unknown**	**frozen muscle**	**MPCR + direct sequencing**	**LC049953**
	**EO2**	**unknown**	**#35**	**Male**	**unknown, China**	**frozen muscle**	**MPCR + direct sequencing**	**LC049378**
	**EO3**	***L*. *l*. *chinensis***	**#39**	**Male**	**Sichuan, China**	**frozen muscle**	**MPCR + direct sequencing**	**LC049952**
	**EO4**	**unknown**	**#52**	**Female**	**unknown, China**	**frozen muscle**	**Long range PCR + direct sequencing**	**LC049377**
	**EO5**	**unknown**	**NMNS-CA209**	**unknown**	**Sakhalin, Russia**	**dried tissue**	**MPCR + direct sequencing**	**LC049954**
**Japanese otter**	**JO1**	**unknown**	**YCM-M0001**	**unknown**	**Kanagawa, Japan**	**dried tissue**	**Illumina**	**LC049955**
	**JO2**	**unknown**	**NZP-SS-01**	**Male**	**Kochi, Japan**	**inner part of paw pad**	**Illumina**	**LC050126**
	**JO3**	**unknown**	**unknown**	**unknown**	**Fukushima, Japan**	**dried tissue**	**Illumina**	**undetermined**

The studbook numbers come from the Internal Studbook of the Eurasian Otter (*Lutra lutra*) for the Japanese Association of Zoos and Aquariums (JAZA). NMNS-CA: National Museum of Nature and Science, Comparative Anatomy collections. YCM-M: Yokosuka City Museum, Mammal. NZP-SS: Noichi Zoological Park, Stuffed Specimen.

To obtain whole mtGenome sequences, we employed the Multiplex PCR (MPCR) method [16]. Using the MPCR method, we determined the complete mtGenome sequences of four Eurasian otters: EO1–EO3 and EO5. To obtain amplicons of MPCR fragments, we divided the mtGenome into 46 fragments with overlapping regions in neighboring fragments (S1 Fig). To amplify the 46 fragments, outer (for MPCR) and inner (for simplex PCR) primers were designed for each fragment. In total, we designed 167 primers for this method according to the complete mtGenome sequence of a South Korean Eurasian otter (GenBank accession No. FJ236015) (S1 Table). The amplicons varied in length from 408 to 545 bp (including primers) and they covered the entire mtDNA of the Eurasian otter (S1 Fig for schematic representation and S1 Table). We used two sets of outer primers to amplify the *L*. *lutra* mtGenome by MPCR. Set 1 comprised primers for odd numbered fragments (23 primer pairs). Set 2 comprised primers for even numbered fragments (23 primer pairs) (S1 Table). The two MPCR assays comprised 1 U Ex *Taq* polymerase (TaKaRa, Japan), 1× Ex *Taq* buffer, 0.4 mM dNTPs, 1 μM of each primer, and 1–50 ng of genomic DNA in a final volume of 50 μL. We included negative controls in each set of amplifications to check for contamination. The MPCR conditions were as follows: 27 cycles at 94°C for 20 s, 50°C for 30 s, and 72°C for 1 min. The MPCR amplification products were used as templates for each of 23 simplex amplifications. During simplex (second) amplification, we used the inner PCR primers (S1 Table). These were the internal primers of those used in MPCR. The simplex PCR assay comprised 0.5 U Ex *Taq* polymerase, 1× Ex *Taq* buffer, 0.4 mM dNTPs, 1 μM of each primer (single primer pair), and 1 μL of the MPCR products in a final volume of 25 μL. The simplex PCR temperature profile was identical to that described above, except that there were 33 cycles instead of 27. The MPCR and simplex PCR products were confirmed by electrophoresis on a 1.5% Agarose S (Nippon Gene, Japan) gel and stained with ethidium bromide. Sequencing was performed using an ABI BigDye Terminator v3.1 Cycle Sequencing kit (Thermo Fisher Scientific, USA) with an ABI Applied Biosystems 3500 Genetic Analyzer, or sequencing was outsourced to Macrogen Japan. The reactions comprised 0.5 μL BigDye ver. 3.1 terminator premix, 1× sequencing buffer, 1 μM sequence primer, and 1 μL of the PCR product in a final volume of 5 μL. The sequencing primers were the same as the simplex PCR primers (inner primers). The sequencing conditions used for PCR were as follows: 25 cycles at 96°C for 15 s, 50°C for 15 s, and 60°C for 2 min.

The whole mtGenome of one Eurasian otter (EO4) was amplified in two fragments using the long range PCR technique [17]. The long range PCR primers and nested PCR primers were designed on the basis of conservation across the mtGenomes of Mustelidae (Mammalia, Carnivora). Four primers were used for long range PCR and 14 primers were used for nested PCR (S2 Fig and S2 Table). We designated the two fragments obtained from the long range PCR assay as L1 and L2 (S2B Fig). The long range PCR amplification protocol was as follows: 94°C for 1 min, followed by 30 denaturation cycles at 98°C for 10 s, and then annealing and extension at 68°C for 15 min. The reactions comprised 1 U KOD FX Neo (TOYOBO, Japan), 1× PCR Buffer for KOD FX Neo, 0.4 mM dNTPs, 0.3 μM of each primer, and 100 ng of genomic DNA in a final volume of 50 μL. The PCR amplifications were cleaned using ExoSAP-IT (Affymetrix/USB, USA) prior to the nested PCR amplification. To obtain templates for direct sequencing of the mtGenome, the long range PCR products were employed as templates for nested amplification, which were then used for direct sequencing. We amplified four different fragments (L1-N1, L1-N2, L1-N3, and L1-N4) of mtDNA from the L1 fragment template and three different fragments (L2-N1, L2-N2, and L2-N3) of mtDNA from the L2 fragment template (S2 Fig and S2 Table). The PCR protocol used to amplify these seven fragments was as follows: 30 cycles of denaturation at 94°C for 45 s, annealing at 50°C for 45 s, and extension at 72°C for 3.5 min. The PCR mixture comprised 0.5 U Ex *Taq* polymerase, 1× Ex *Taq* buffer, 0.4 mM dNTPs, 1 μM of each primer, and 1 μL of each long range PCR product in a final volume of 25 μL. We included negative controls in each long range PCR and nested PCR amplification set to check for contamination. The long range PCR and second PCR products were confirmed by electrophoresis on a 1.0% Agarose S gel and stained with ethidium bromide. The sequencing PCR conditions were as follows: 25 cycles at 96°C for 15 s, 50°C for 15 s, and 60°C for 2 min. The sequencing primers are shown in S1 Table.

Editing and contig assembly for the mtDNA sequences were performed using Genetyx ver.12 (Genetyx Corporation, Japan) and were carefully checked by eye.

### Samples of Extinct Japanese Otter

To prevent ancient DNA from being contaminated by modern DNA, the laboratory within which DNA from Japanese otters was processed was completely separate from that where the DNA of the modern Eurasian otters were processed. To avoid contamination, DNA was extracted from the three extinct Japanese otters on a class 100 clean bench (MCV-B131F; Sanyo, Japan) at the Tokyo University of Agriculture. Before extraction, we sterilized the clean bench using ultraviolet radiation for at least an overnight. Subsequently, the library was constructed on a class 100 clean bench (MCV-131BNS; Sanyo, Japan) at the National Institute of Polar Research. Before constructing the library, we sterilized the clean bench using ultraviolet radiation for at least an overnight. All procedures mentioned above were performed in a separate room from that containing the thermal cyclers and PCR products. All equipments used for DNA extractions were cleaned before use using bleach (5%) or DNA Away (Molecular BioProducts, USA).

We collected three specimens of Japanese otters. Japanese otter 1 (JO1) was captured in Jogashima, Misaki-cho, Miura, Kanagawa Prefecture, during 1915 or 1916 (Table 1 and Fig 1A). JO1 specimen is stored at the Yokosuka City Museum located in Fukadadai, Yokosuka, Kanagawa prefecture, Japan. Japanese otter 2 (JO2) was captured in Akadomari, Otsuki-cho, Hatagun, Kochi Prefecture, in 1977 (Table 1 and Fig 1B). JO2 specimen is stored at NZP located in Otani, Noichi-cho, Konan-shi, Kochi prefecture, Japan. Japanese otter 3 (JO3) was captured in Sukagawa, Fukushima Prefecture during 1935 (Table 1 and Fig 1A). JO3 specimen is stored at the Agriculture and Forestry Research Institute, Shizuoka Prefecture located in Negata, Hamakita-ku, Hamamatsu-shi, Shizuoka prefecture, Japan. However, JO3 specimen was not assigned the number of specimen voucher. We obtained tissue samples for DNA extraction from these three specimens using dried muscle tissue in JO1 and JO3, and from the inner part of the paw pad in JO2. DNA was extracted from these Japanese otters prepared as described above, using the DNeasy Blood & Tissue kits (Qiagen, Netherlands). In this experiment, we employed a modified protocol as follows: 1) the samples were incubated with rotation at 56°C for 6 h; 2) 20 μL of proteinase K solution was added to the sample, which was incubated with rotation at 56°C for 6 h, and; 3) DNA elution was repeated twice using 100 μL of AE buffer from the spin column (total volume = 200 μL).

We attempted to amplify mtGenomes of Japanese otters using the MPCR method as describe above; however, we obtained only a few fragments using this procedure. Therefore, we chose to determine mtGenome sequences of Japanese otters using the MiSeq desktop sequencer.

To construct the sequencing libraries, a NEBNext Ultra DNA Library Prep kit for Illumina (New England Biolabs, USA) was used for the Japanese otter DNA, according to the manufacturer’s instructions with the exception of PCR cycles, which was replace by 15 cycles of PCR. The amplified library products were isolated in agars gels (size of 200–700 bp) and purified by use of NucleoSpin Gel and PCR Clean-up (TaKaRa, Japan). We diluted the three sequencing libraries of JO1–JO3 equimolarly. The three libraries were used as a template for paired-end sequencing using one lane of a MiSeq reagent kit v3 and a MiSeq desktop sequencer (Illumina, USA).

Read files (fastq.gz) were generated using MiSeq Reporter software version 2.3.32 (Illumina, USA). We applied the “Remove Duplicate Reads” function in the CLC Genomics Workbench version 7.5.1 (Qiagen, Netherlands). The sequencing data were then trimmed after removing the duplicate reads, where the trim function used the following parameters: ambiguous limit = 3, quality limit = 0.01, remove 5′ nucleotide = 1 bp, and remove 3′ nucleotide = 1 bp; and collected 30–200-bp reads. The *de novo* assembly function was used to assemble the trimmed reads. The assembly process used the following parameters: mismatch cost = 2, insertion cost = 3, deletion cost = 3, length fraction = 0.98, and similarity fraction = 0.98. Only contigs >1,000 bp were retained. To determine the mtGenome length (approximately 16,400 bp), the contigs were searched using BLAST (http://blast.ncbi.nlm.nih.gov/Blast.cgi).

The CLC Genomics Workbench “Reference to Mapping” function was used to assemble the trimmed reads with the reference *L*. *lutra* mtGenome, where the control region (CR) was translocated to the 5′ terminal (GenBank accession No. LC049952). The assembly process used the following parameters: mismatch cost = 2, insertion cost = 3, deletion cost = 3, and length fraction and similarity = 0.9. The paired-end reads that mapping to multiplace in the reference were mapped using random settings.

### mtGenome Annotation

Transfer RNA (tRNA) and ribosomal RNA (rRNA) genes were identified, and their secondary structures were estimated using the MITOS web server [18]. Protein-coding regions, CR, L-origin, and non-coding regions were identified manually by referring to the previously reported entire mitochondrial sequences of Eurasian otters (EF672696 and FJ236015).

### Phylogenetic Analyses and Divergence Time Estimation

Previously, Koepfli and Wayne [1] and Koepfli et al. [10] characterized the partial *ND5* gene (692 bp) and complete *cytb* gene (1,140 bp) in species of Lutrinae living in Asia. Based on their results, we determined the molecular characteristics of Japanese otters by comparing them with those of *L*. *lutra*, *L*. *sumatrana*, *Lutrogale perspicillata*, and *A*. *cinerea*, which are distributed sympatrically in Asia. We compared the partial *ND5* gene and complete *cytb* gene sequences of 11 species of Lutrinae (S3 Table). Furthermore, we included the partial *cytb* gene sequences (224 bp) of a Japanese otter reported by Suzuki et al. [10] in this comparative analysis (S1 File). We also computed the pairwise distance on the basis of Kimura’s 2-parameter (K2P) model [19] after pairwise deletion of missing data from *cytb* by MEGA 6.06 [20].

We performed two dataset phylogenetic analyses as follows. To determine the phylogenetic position of the Japanese otter in the Lutrinae clade, we obtained mitochondrial partial *ND5* gene (692 bp) and complete *cytb* gene (1,140 bp) sequence data from 11 species of Lutrinae in GenBank (S3 Table). In addition, we added the *L*. *nippon* (Ehime) sequence to this analysis according to Suzuki et al. [10] (S1 File). *Pteronura brasiliensis* (giant otter) was used as outgroup in this analysis. We removed start/stop codons from each sequence in the *ND5* + *cytb* dataset. The total sequence length of the *ND5* + *cytb* dataset was then 1,826 bp. Another analysis aimed to elucidate the phylogenetic position of Japanese otters in the clade of the genus *Lutra* according to the mtGenome dataset (S3 Table). *Enhydra lutris* (sea otter) was used as outgroup in this analysis (S3 Table). We removed non-coding regions, start/stop codons, CR, L-origin, overlapping regions [between *ATP6* and *ATP8*, *ND4* and *ND4L*, *ND5* and *ND6*, tRNA-Ile (AUY) and tRNA-Gln, tRNA-Leu (CUN) and *ND5*, and tRNA-Thr and tRNA-Pro], and the *ND6* gene from the mtGenome dataset. The *ND6* gene is the only protein-coding gene coded on the L strand; thus, it has quite different evolutionary properties compared with those of the other 12 protein genes [21]. The resulting total sequence length in the mtGenome dataset was 14,740 bp.

The sequence datasets were aligned automatically using MAFFT ver.7.21 [22] with the G-INS-i option and they were carefully checked by eye. We inferred neighbor-joining (NJ) and maximum likelihood (ML) trees from the two datasets. NJ analyses were performed using MEGA 6.06 [20] with the K2P model and Gamma distribution. The confidence of the internal nodes was evaluated by bootstrapping with 1000 replicates. ML analyses were conducted using RAxML v8.1.1 [23,24] with the GTR+Γ+I model [25–27].

By considering the differences in the tempo and modes of the genes, two datasets were distinguished as follows. The *ND5* + *cytb* gene dataset was separated into three partitions: first codon, second codon, and third codon positions. The other dataset was separated into five partitions: 22 tRNAs, two rRNAs, and first codon, second codon, and third codon positions. Bootstrapping was applied with 1000 replicates. All gaps were treated as missing data. We then constructed the consensus relationships on the basis of these analyses.

We estimated divergence time among the species of Carnivora. The complete mitochondrial protein sequences of 73 species of Carnivora and one species of Pholidota as well as the complete *cytb* sequences of eight species of Lutrinae were downloaded from NCBI; the accession numbers are shown in S4 Table. All 12 protein-coding genes in the H strand of the mtGenome were aligned separately using the MUSCLE program [28] implemented in MEGA 6.06 and carefully checked by eye. The start codons, stop codons, and overlapping regions (between *ATP6* and *ATP8*, *ND4* and *ND4L*, and *ND5* and *ND6*) were excluded and concatenated into a single alignment measuring 10,704 bp in length. Eight Lutrinae species had data only for *cytb;* therefore, the other 11 genes were treated as missing data for these eight species. A phylogenetic tree was inferred by RAxML 7.2.8 [24] using the GTR+Γ+I model. A partition model was employed that considered the difference among the three codon positions. To evaluate the confidence of the internal branches, bootstrapping was applied using the rapid bootstrap algorithm [24] with 1000 replicates.

The divergence times were estimated on the basis of the ML tree topology using the MCMCTREE program of PAML 4.7 [29]. The mitochondrial sequence data mentioned above were used, but *cytb* and the other 11 genes were treated as different datasets. Sasaki et al. [30] demonstrated the superior performance of the codon substitution model [31] compared with normal nucleotide substitution models such as the GTR+Γ+I model. Naturally, it is expected that the accuracy of the estimated branch lengths is related directly to the accuracy of the time estimation; therefore, a codon substitution model was employed in this analysis. The normal approximation method was used to reduce the computational burden. The independent rate model [32] performs better than the auto-correlated model [33] in terms of the Bayesian factor; thus, the former model was applied. MCMC was conducted in the following conditions. The prior distribution of the root rate was (4, 0.588) and σ^2^ was (1 0.7). The total generation length was 4,000,000 and trees were sampled every 200 generations. The first 200,000 generations were discarded as a burn-in. The calibration points based on fossil records were the same as those described in our previous studies [34,35]: the divergence time between Mustelidae and Procyonidae was assumed to be older than 28.5 Ma based on the oldest procyonid species *Pseudobasaaris*; the emergence of the crown Phocidae was assumed to be older than 14.5 Ma based on the oldest crown phocid species *Monotherium wymani*; the emergence of the crown Pinnipedia was assumed to be older than 21.5 Ma based on the oldest crown pinniped species *Desmatophoca brachycephala* and younger than 28.6 Ma based on the oldest stem pinniped species *Enaliarctos tedfordi*; the emergence of the crown Arctoidea was assumed to be older than 39.6 Ma based on the oldest ursoid species *Amphicyon* sp; and the emergence of the crown Carnivora was assumed to be older than 43 Ma based on the oldest crown carnivoran species *Tapocyon* and younger than 63.8 Ma based on the oldest stem carnivorans (miacids). Convergence of the parameters was confirmed using the TRACER ver. 1.5 program (http://tree.bio.ed.ac.uk/software/tracer/) by checking that all of the effective sample sizes exceeded 200.

The divergence time in the genus *Lutra* was also estimated on the basis of the mtGenome sequence data. Ho et al. [36] demonstrated that the time dependency of the evolutionary rate is higher late in the short term (<1–2 Ma) and lower late in the long term (>1–2 Ma). This is probably because slightly deleterious mutations are not eliminated completely from populations within a short evolutionary period. Therefore, it is preferable to use nearly neutral evolving sites for divergence time estimation at this time scale. Endicott and Ho [37] indicated good performance of the third codon positions in this case. Thus, we applied only the third codon position in the complete mitochondrial protein genes. *E*. *lutris* and *L*. *sumatrana* (*cytb* only) were used as outgroup.

Only ten sequences were included in this analysis; therefore, the likelihood function was estimated exactly during the MCMC process. The prior distribution of the root rate was (4, 1.42) and σ2 was (1 0.107). The total generation length was 5,000,000 and trees were sampled per 50 generations. The first 100,000 generations were discarded as a burn-in. The divergence times of *Enhydra*–*Lutra* (9.0–12.5 Ma) and *L*. *sumatrana*–*L*. *lutra* (1.8–4.8Ma) were inferred from the complete mitochondrial protein genes in the framework of all Carnivora, and they were used as calibration points in this analysis.

## Results and Discussion

### Comparison with Previously Studied Sequences

Using MPCR and long range PCR, we determined five nearly complete mtGenomes of Eurasian otters (Table 1). In the CR of all mtGenomes, the Eurasian otter possessed tandem repeats, as reported in previous studies [38,39]. It was previously shown that the repeating unit in the Eurasian otter comprises from two to ten repeats of 5′-CAC GTA CGY AYA CAC GCA CAC B-3′. In the present study, we detected eight or ten repeats in five Eurasian otters. However, the repeat number was not accurate according to sequence determination using the Sanger method, which was assumed to be due to template slippage during the PCR process [40]. Therefore, we excluded the tandem repeats from the total length of the mtGenome. The length of each specimen was as follows: 16,316 bp for EO1 (unknown), EO4 (China), and EO5 (Sakhalin, Russia) and 16,317 bp for EO2 (China) and EO3 (Sichuan, China). The detailed composition of each specimen’s mtGenome is shown in S5 Table.

We obtained 140 contigs from JO1 and 44 contigs from JO2 by applying the read data obtained from NGS to the *de novo* assembly of the Japanese otter samples. Both contigs had one contig with a length similar to the mammalian mtGenome (approximately 16,400 bp), which were broadly similar to the mtGenomes of *L*. *lutra* and *E*. *lutris* according to BLAST. Although we obtained 463 contigs from JO3, there was no contigs that were similar to the mtGenomes of *L*. *lutra*.

The contig of JO1 generated on the basis of 157,703 reads had a length of 16,316 bp (excluding tandem repeats), and the contig of JO2 generated on the basis of 22,187 reads had a length of 16,319 bp (excluding tandem repeats) (S6 Table). In addition, by mapping to reference, we obtained consensus sequences for JO1 and JO2 with lengths of 16,316 bp (172,071 reads) and 16,319 bp (23,410 reads), respectively (excluding tandem repeats) (S6 Table). The depth of coverage for these contigs was more than 100× (S6 Table). On the other hand, we could only map 146 reads to the *L*. *lutra* mtGenome in JO3. The depth of coverage for this data was only 0.96×. Hence, we could not determine the mtGenome from JO3 specimen in this study.

All seven of the mtGenomes determined in this study had the general mammalian mtGenome structure (13 protein-coding genes, 22 tRNAs, two rRNAs, CR, and L-origin; S5 Table). There were no internal stop codons in the protein-coding region. Therefore, we consider that all specimens had been sequenced successfully. The sequence data for these mtGenomes have been deposited in GenBank (accession Nos. LC049377, LC049378, LC049952–LC049955, and LC050126).

Previously, the mtGenomes of Lutrinae species were determined in two Eurasian otters (GenBank accession Nos. EF672696 and FJ236015) and one sea otter (GenBank accession No. NC_009692). We compared our seven mtGenome sequences from Eurasian and Japanese otters with the existing sequences of two South Korean otters. The lengths of all the mtGenomes ranged from 16,316 bp to 16,319 bp, except for a South Korean otter (EF672696) where we confirmed a five-amino acid sequence deletion in *COX3* and a four-amino acid sequence deletion in the *ND6* gene even when we compared it with the mtGenome of the sea otter (S3 Fig). On the basis of these results, we considered that the data for EF672696 contained measurable artificial sequencing errors. Therefore, we excluded the data for EF672696 from our subsequent analysis.

**Fig 2:**
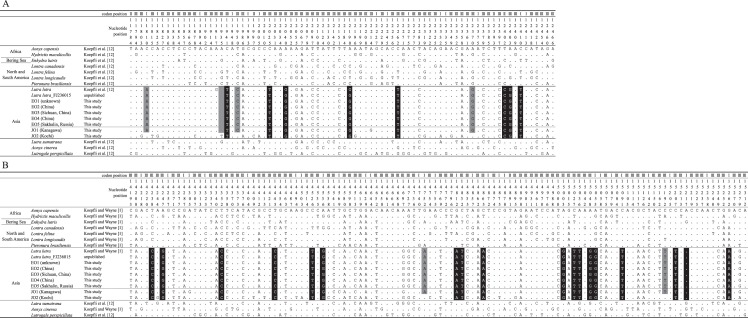
Diagnostic nucleotides in the *ND5* and *cytb* genes compared with those used in previous studies. (A) Partial *ND5* gene (692 bp). (B) Complete *cytb* gene (1,140 bp). Nucleotide positions that are identical to those in the *Aonyx capensis* sequence are denoted by a period (.). The nucleotide position numbers are based on a Eurasian otter from China (EO4). Letters highlighted in black indicate two Japanese otters (JO1 and JO2) shared diagnostic nucleotides with the Eurasian otter. The sites highlighted in gray indicate the diagnostic nucleotides shared by JO1 (Kanagawa) and the Eurasian otter.

To estimate the genetic identity of the two Japanese otters, we compared the genetic data for JO1 and JO2 with those for *L*. *lutra*, *L*. *sumatrana*, *Lutrogale perspicillata*, and *A*. *cinerea*, which live sympatrically in Asia. Previously, Koepfli and Wayne [1] and Koepfli et al. [12] characterized the partial *ND5* gene (692 bp) and complete *cytb* gene (1,140 bp) in Asian Lutrinae. On the basis of their results, we characterized molecular diagnostics for the Japanese otter. Among the Asian Lutrinae, the sequences of the two Japanese otters (JO1 and JO2) were identical with the diagnostic nucleotides of *L*. *lutra* at 26 sites, as shown in Fig 2 (highlighted in black). Furthermore, JO1 had additional six diagnostic nucleotide sites of *L*. *lutra*, which indicated its close genetic relationship to *L*. *lutra* (Fig 2, highlighted in gray). In contrast, JO2 shared only three diagnostic nucleotide sites with *L*. *sumatrana* (nucleotide positions 11815, 12332, and 14244 in Fig 2) and one site with *Lutrogale perspicillata* (nucleotide position 12319 in Fig 2). In addition, each Japanese otter shared a single diagnostic nucleotide with *A*. *cinerea* in different nucleotide positions (nucleotide position 14277 in JO2 and 14740 in JO1). Hence, most of the diagnostic nucleotide sites observed in Japanese otters were shared with *L*. *lutra*. This suggests that Japanese otters are relatively closely related to *L*. *lutra* among the Asian Lutrinae.

### Phylogenetic Position of Japanese Otter in the Lutrinae Lineage

**Fig 3:**
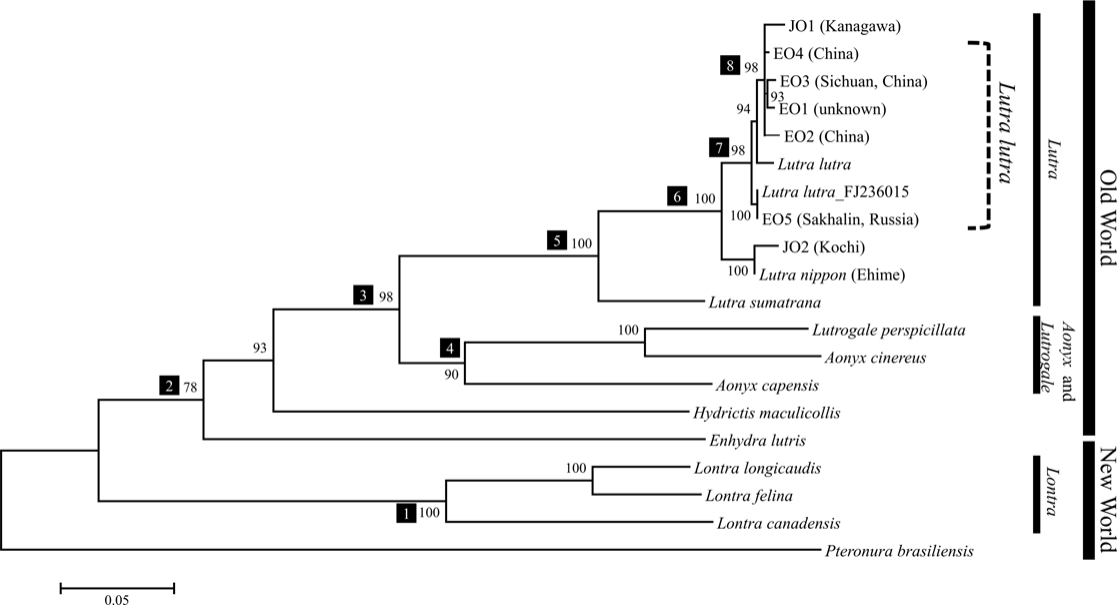
Phylogenetic tree for Lutrinae based on the partial mtDNA together with the *L*. *nippon* (Ehime). This ML tree was based on the partial *ND5* gene (692 bp) and complete *cytb* gene (1,134 bp) dataset, which included the *L*. *nippon* (Ehime) sequence (224 bp) [10]. This tree was estimated using the GTR+Γ+I model. Numbered boxes denote nodes. The nodal number indicates the BP value. BP was estimated on the basis of 1,000 bootstrap replicates. The evolutionary constraints on the nucleotide substitutions must differ between the first, second, and third codon positions; therefore, we specified partitions for each region. OTUs without localities are previously reported data (Koepfli and Wayne [1]; Koepfli et al. [12]). Data for *L*. *lutra* (South Korea) is FJ236015.

Previously, Koepfli et al. [12] reported the phylogenetic relationships among 11 species of Lutrinae excluding the Japanese otter. Using their sequence data, we constructed an ML tree to estimate the phylogenetic position of the Japanese otter in the clade of Lutrinae (Fig 3). In this analysis, *P*. *brasiliensis* (giant otter) was used as outgroup. According to the results, the topology of the tree obtained was consistent with that presented by Koepfli et al. [12]. The Japanese otters were included in the Old World clade and they formed a monophyletic group with Eurasian otters with strong statistical support [100% bootstrap probability (BP); Fig 3, node 6]. Therefore, we conclude that the Japanese otter is a species or subspecies in the genus *Lutra* and is most closely related to *L*. *lutra* in this genus. This opinion agrees with the results of previous morphological and molecular phylogenetic studies [8–10]. Imaizumi and Yoshiyuki [8] reported that the rhinarium and nostril pad of the Japanese otter are entirely naked, as found in *L*. *lutra*, but unlike the hair-covered rhinarium observed in *L*. *sumatrana* [2]. In the *L*. *lutra* + Japanese otter clade, JO1 represented an intraspecific group relative to *L*. *lutra*, where it formed a subclade with three individuals from China containing *L*. *l*. *chinensis* (EO3), and one individual from an unknown locality (99% BP; Fig 3, node 8). Previously, Koepfli et al. [12] characterized the range of intraspecific genetic distances among 41 specimens of *L*. *lutra* derived from Europe, the Middle East, and Far East as 0.05%–1.15%. In the present study, the range of genetic distances between JO1 and *L*. *lutra* individuals was 0.6%–1.2% (S6 Table). The genetic distances observed in JO1 belonged mostly to the intraspecific range. Thus, we suggest that this individual should be classified as *L*. *lutra*. However, the phylogenetic position of JO2 differed from that of JO1, although they were both described as Japanese otters. The JO2 lineage formed a sister to the clade comprising *L*. *lutra* and JO1 (98% BP; Fig 3, node 7). The range of genetic distances between JO2 and *L*. *lutra* individuals, including JO1, was 2.4%–3.3% (S7 Table), and it did not overlap with the intraspecific distances. Moreover, the genetic distance between JO2 and *L*. *sumatrana* was 7.2% (S7 Table). Similarly, the range between *L*. *lutra* individuals, including JO1, and *L*. *sumatrana* was 6.7%–7.4% (S7 Table). Hence, JO2 was clearly diverged genetically from *L*. *sumatrana*. However, we did not use specimens of European Eurasian otters (*L*. *l*. *lutra*) in this study. Thus, it is possible that the JO2 lineage represents a lineage of the subspecies *L*. *l*. *lutra* that lives in Europe. Previous studies have reported the population structures of *L*. *l*. *lutra* using more than 500 individuals on the basis of the partial mitochondrial CR [41–43]. Therefore, we used the data from these studies to calculate the time of the most recent common ancestor (tMRCA) of the *L*. *lutra* population according to the mitochondrial CR using the coalescent method. In this analysis, we assumed two populations of *L*. *lutra*, as follows: a Eurasian *L*. *lutra* population that comprised Eurasian *L*. *lutra* population (*L*. *l*. *lutra* and East Asian *L*. *l*. *chinensis* + JO1), and a Eurasian + JO2 population that comprised the Eurasian *L*. *lutra* population and JO2 (S2 File). The tMRCAs of the Eurasian *L*. *lutra* population and the Eurasian + JO2 population were calculated as 282,200 and 498,300 years ago, respectively. Therefore, the tMRCA of the Eurasian + JO2 population was older than that of the Eurasian *L*. *lutra* population (S4 Fig). The 95% confidence intervals of the coalescent times of these two populations overlapped (S4 Fig), but the coalescent time of the Eurasian + JO2 population was significantly older according to a two-sample *t*-test (*P* < 0.01). This result shows that the JO2 lineage clearly differed from the species of *L*. *lutra* living in Europe and Asia, i.e., *L*. *l*. *lutra* and *L*. *l*. *chinensis*. Therefore, we consider that JO2 should be classified as an independent species or subspecies in the genus *Lutra*. According to this result, we performed more intensive phylogenetic analyses using mtGenome sequences.

### Phylogenetic Relationships among Japanese and Eurasian Otters

**Fig 4:**
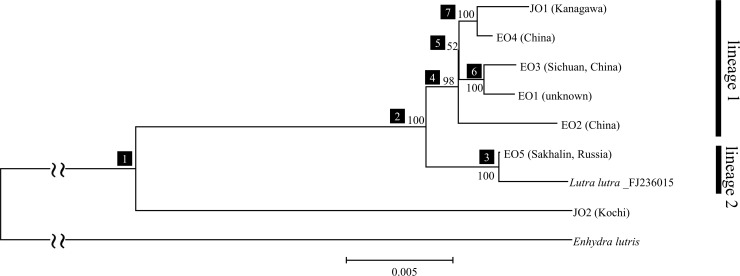
Phylogenetic tree of Eurasian and Japanese otters based on the mtGenome. The ML tree based on the mtGenome comprised 14,740 bp (tRNAs = 1,488 bp, rRNAs = 2,532 bp, and protein-coding genes = 10,720 bp). This tree was estimated using the GTR+Γ+I model. Numbered boxes denote nodes. The nodal number indicates the BP value. BP was estimated on the basis of 1,000 bootstrap replicates. The evolutionary constraints on nucleotide substitutions must differ between the first, second, and third codon positions as well as between tRNA and rRNA. Therefore, we specified partitions for each region. Data for the *E*. *lutris* and *L*. *lutra* (South Korea) were reported previously NC_009692 and FJ236015, respectively.

To elucidate the detailed phylogenetic relationships among the individual Japanese otters and *L*. *lutra*, we constructed an ML tree on the basis of the mtGenome and the tree obtained is shown in Fig 4. *E*. *lutris* (sea otter) was used as outgroup in this phylogenetic analysis. In this tree, Eurasian otters and JO1 formed a monophyletic group with 100% BP, which diverged into two lineages (lineages 1 and 2 in Fig 4). JO1 formed a monophyletic group with EO4 (China) (100% BP; Fig 4, node 7) and it was included in lineage 1 with EO1 (unknown), EO2 (China), and EO3 (Sichuan, China; *L*. *l*. *chinensis*) (98% BP; Fig 4, node 4). Lineage 2 comprised Eurasian otters from South Korea and EO5 (Sakhalin, Russia). Our results suggest that genetically two diverged lineages exist among the Eurasian otters living in East Asia. Moreover, one of the Japanese otters, i.e., JO1, was obviously included in lineage 1. Therefore, we identified JO1 as *L*. *lutra*.

We confirmed that JO2 had a long independent evolutionary history in *Lutra* and that JO2 was in a sister clade of other *L*. *lutra*, including JO1, as suggested by the phylogeny of the partial *ND5* and *cytb* genes shown in Fig 3 (Fig 4, nodes 1 and 2). The phylogenetic status of JO2 shown in Fig 4 is consistent with that reported by Suzuki et al. [10] who determined a partial *cytb* sequence (224 bp) from an individual Japanese otter captured in Ehime Prefecture (Shikoku Prefecture, Fig 1B) during 1962 and four Eurasian otters (*L*. *l*. *lutra*, *L*. *l*. *chinensis*, and an unknown locality). According to their analysis, the Japanese otter was in a sister taxa relative to a monophyletic group that comprised four Eurasian otters. When we added the partial *cytb* data reported by Suzuki et al. [10] to our *ND5* + *cytb* dataset, as shown in Fig 3, the Japanese otter described by Suzuki et al. [10] formed a monophyletic group with JO2 with 100% BP (Fig 3). Moreover, the Ehime and Kochi Prefectures are both on Shikoku Island (Fig 1) and the distance between these localities is approximately 20–30 km. The genetic information for the otter from Ehime was represented only by a short partial sequence in the mtGenome, but the otters from Ehime and Kochi probably comprise a distinct lineage in *Lutra*.

### Evolutionary History of the Japanese Otter

To estimate the evolutionary time scale of the two lineages of Japanese otters, we estimated the divergence times among Lutrinae in the framework of all Carnivora. The estimated divergence times among the Carnivora are shown in S5 Fig. In general, these results agree with those obtained in previous studies on the basis of nuclear genes [44,45]. However, when general nucleotide substitution models such as the GTR+Γ model were employed for divergence time estimation, the divergence times were much older (data not shown), which was probably due to underestimation of the number of nucleotide substitutions in the deep branches using the simpler nucleotide substitution model. This suggests that the estimates obtained by the more realistic model were also advantageous for divergence time estimation.

We estimated that *Enhydra* diverged from *Lutra* + *Aonyx* + *Lutrogale* at 10.76 Ma (95%: 9.01–12.49 Ma) and that *L*. *sumatrana* diverged from *L*. *lutra* at 3.24 Ma (95%: 1.80–4.81 Ma) (S5 Fig). These two estimates were then used as calibration points to infer the divergence time of Japanese otters, which showed that the JO2 lineage diverged from the ancestral lineage of Eurasian otters at 1.27 Ma (95%: 0.98–1.59 Ma) during the Early Pleistocene (Calabrian age: 1.80–0.78 Ma) (S6 Fig). The divergence of the JO2 and Eurasian otter lineages is supported by the presence of a land bridge between the Eurasian continent and Japanese islands during the Calabrian age [46]. Indeed, Taruno [47] investigated the fossil record of Proboscidean species that lived on the Japanese islands and suggested that *Mammuthus trogontherii* migrated from the Eurasian continent at 1.2 Ma via this land bridge. In addition, based on a molecular phylogenetic study, it was suggested that the Japanese Asian black bear (*Ursus thibetanus japonicus*), which is an endemic carnivore subspecies in Japan, diverged from the continental subspecies at 1.46 Ma [48]. It is believed that the migration and speciation events in these species are attributable to the formation of a land bridge between the Japanese islands and Eurasian continent near a northern latitude of 32° (after 1.7 Ma) [46]. It is likely that the ancestral population of JO2 also migrated from the Eurasian continent to the Japanese islands using the same land bridge.

In the Japanese islands, Nojima [49] reported the fossil record of a *Lutra* sp. from a 0.3–0.4 Ma stratum in Shizuoka, which is the oldest recorded species related to the genus *Lutra* in the Japanese islands. Thus, it is notable that there is a difference of almost one million years between the molecular divergence time and the fossil record. However, mammalian fossil remains are scarce from the Calabrian age in Honshu, Shikoku, and Kyushu [50,51] because the soil is acidic in Japan, which decomposes animal bones [52]. This may explain why older fossils of *Lutra* sp. have not yet been found.

The divergence time of JO1 was calculated at 0.10 Ma (95%: 0.06–0.16 Ma) during the Late Pleistocene (Tarantian age: 0.126–0.0117 Ma) (S6 Fig), which is a relatively recent event compared with the divergence time of JO2. As similar examples of genetic divergence observed in JO1, Japanese populations of the wild boar (*Sus scrofa leucomystax*) diverged from the continental populations at 0.140–0.253 Ma [53]. Watanobe et al. [53] suggested that ancestor of *S*. *s*. *leucomystax* migrated from the Korean Peninsula to Japan in this period. Therefore, it would appear that the ancestors of JO1 and *S*. *s*. *leucomystax* migrated at same period

However, there is no evidence that a land bridge was present between western Japan and the Asian continent during this period. Meanwhile, Jogashima and the neighboring region where JO1 was caught has been a flourishing base for deep sea fishing for a long time. Thus, it is possible that this specimen was imported from the Asian continent via a deep sea fishing vessel, which may have called at a port on the Asian continent. Therefore, it is also possible that JO1 has been artificially imported from the Eurasian continent to Japan in modern times. Whether JO1 came from a Japanese traditional lineage should be elucidated by further research using more Japanese otter specimens.

## Conclusion

In the present study, we definitively demonstrated that the Japanese otter is a member of the genus *Lutra*, and that the study of the Japanese otters (JO1 and JO2) indicated different evolutionary histories in the clade of *Lutra*. JO2 represents a descendant of an older ancestor who migrated to the Japanese islands during the Early Pleistocene (Calabrian age: 1.80–0.78 Ma). Genetic divergence of JO2 is comparable to the degree of difference of species or of subspecies. On the other hand, our analysis characterized JO1 as a member of *L*. *lutra*. The IUCN Red List treats the taxonomic status of the Japanese otter as under review. Although Imaizumi and Yoshiyuki [8] classified the Japanese otter as *L*. *nippon* as an independent species, the IUCN treats the Japanese otter as a synonym. In contrast to the opinion by Imaizumi and Yoshiyuki [8], even a subspecies name of this animal is not assigned in the IUCN. On the basis of habitat of a subspecies of *Lutra*, the taxonomic status of the Japanese otter may correspond to *L*. *l*. *chinensis*. However, the genetic divergence of JO2 differed considerably from those of *L*. *l*. *chinensis* or *L*. *l*. *lutra*, indicating the possibility of an independent species. Therefore, we propose that researchers should re-examine the taxonomic status of the lineage leading to JO2 with a view to re-classifying the Japanese otter into a subspecies *L*. *l*. *nippon* or independent species *L*. *nippon*. To draw a conclusion to this issue from the viewpoint of molecular phylogenetic study, we should analyze the genetic divergence and phylogenetic relationships among all subspecies of *L*. *lutra*, as we currently only recognize two subspecies (*L*. *l*. *lutra* and *L*. *l*. *chinensis*). In addition, to estimate the genetic divergence of the population level of the Japanese otter, we should use more than one specimen of each lineage of the Japanese otter. Currently, we use only one specimen in each lineage. Moreover, phylogenetic analysis on the basis of genetic information in nuclear DNA is an important direction for future research.

## Supporting Information

S1 Fig Map of the otter mtGenome. (A) Map of the otter mtGenome. (B) Positions of the outer primers, inner primers, and common primers. Circular genome (orange) showing the positions of two rRNAs, 13 proteins, 22 tRNAs (green boxes), and CR. Red (even number) and blue (odd number) frames indicate Set 1 and Set 2 of MPCR amplicons, respectively.
(TIF)

S2 Fig Genome organization and amplification strategy for the otter mtGenome. (A) 12 protein-coding genes are encoded by the H strand, but only the ND6 gene is encoded by the L strand. The 22 transfer RNAs are designated by single-letter amino acid codes. The RNAs encoded by the H strand and L strand are shown above and below the mtGenome maps, respectively. (B) Two segments (L1 and L2 fragments) that covered the mtGenome were amplified with two pairs of long range PCR primers. (C) To obtain templates for direct sequencing, nested PCR was performed using the long range PCR products as templates. Seven pairs of nested PCR primers amplified seven fragments (L1-N1, L1-N2, L1-N3, L1-N4, L2-N1, L2-N2, and L2-N3) that covered the mtGenome.
(TIF)

S3 Fig Indel variable sites observed in Eurasian and Japanese otters. Dashes indicate gaps in the sequence alignment. *E*. *lutris* (sea otter) [34] is shown at the bottom of the alignment for comparison. The numbers of the nucleotide positions are based on the nucleotide positions in the Eurasian otter (GenBank accession No. LC049377), except for the numbers marked by asterisks. The numbers marked by asterisks are based on the nucleotide positions defined in the South Korean Eurasian otter EF672696 [39].
(TIF)

S4 Fig Dynamics of the population size and tMRCAs of Eurasian and Japanese otters. The posterior probability distribution of the tMRCA was inferred from the mitochondrial control regions using the coalescent method with the BEAST program. The vertical axis indicates the posterior probabilities and the horizontal axis indicates the tMRCA (as years before present). The posterior probability distribution colored in blue denotes the population of Eurasian otters excluding JO2, and the distribution colored in black denotes the population including JO2.
(TIF)

S5 Fig Divergence time of Carnivora estimated by the codon model. The nodal number indicates the estimated divergence time. Horizontal dark gray bars show the 95% credibility interval for the divergence time. Numbered boxes denote nodes.
(TIF)

S6 Fig Divergence time of Japanese and Eurasian otters. The nodal number indicates the estimated divergence time. Horizontal dark gray bars show the 95% credibility interval for the divergence time. Numbered boxes denote nodes.
(TIF)

S7 Fig Variable sites observed in the *cytb* sequence of 11 species of Lutrinae and three *cytb* clones of the Japanese otter (Ehime). Nucleotide positions that are identical to those in the *Aonyx capensis* sequence are denoted with a period (.). Dashes indicate gaps in the sequence alignment. The numbers of the nucleotide positions are based on the nucleotide positions in the Eurasian otter (FJ236015). Letters highlighted in black indicate deletion site in the ps7. Letters highlighted in gray indicate the mutation sites between the c5 and the c4. Letters boxed by bold line indicate the mutation sites between seven individuals of *L*. *lutra* and the c4 + JO2.
(TIF)

S1 File Estimation of an ortholog of the *cytb* gene in the Japanese otter (Ehime). (DOCX)

S2 File Coalescent analysis of Eurasian otters on the basis of mitochondrial DNA data. (DOCX)

S3 File Esri permission. (DOCX)

S1 Table MPCR and simplex PCR primers used for the amplification of otter mtGenome. Asterisks (*) indicate use of nested PCR product sequencing.
(XLSX)

S2 Table Long range PCR primers and nested PCR primers for Mustelidae. Asterisks (*) indicate the use of long range PCR.
(XLSX)

S3 Table Mitochondrial DNA data used for phylogenetic analysis in this study. (XLSX)

S4 Table Mitochondrial DNA data used for divergence time estimation in this study. (XLSX)

S5 Table The construct of the mtGenome of Eurasian and Japanese otters. (XLSX)

S6 Table Number of NGS reads, *de novo* assembly result, and mapping to reference result. a) The number of reads after quality trim by CLC genomic workbench. b) Using otter mtGenome contig. c) Average length of otter mtGenome contig reads. d) Average coverage of otter mtGenome contig. e) Using the consensus sequence of mapping. f) Average length of mapping reads. g) Average coverage of mapping consensus sequence. h) Including tandem repeat length.
(XLSX)

S7 Table Pairwise genetic distances among the species or individuals of Lutrinae based on the *cytb* gene (1,140 bp). This table show pairwise distances (genetic distances) that were estimated by the K2P model, pairwise deletion of missing data, and inclusion of all codon positions and substitution types from *cytb*.
(XLSX)

## Abbreviations

BP: bootstrap probability
CR: Control Region
IUCN: International Union for Conservation of Nature
K2P: Kimura’s 2-parameter
Ma: Mega annum
mtGenome: mitochondrial genome
ML: Maximum Likelihood
MPCR: Multiplex PCR
NGS: high-throughput, “next-generation” DNA sequencing
NJ: neighbor-joining
NZP: Noichi Zoological Park
PCR: polymerase chain reaction
tMRCA: time of the Most Recent Common Ancestor
