# Supplementary figures and images for: Evaluating the Phylogenetic Status of the Extinct Japanese Otter on the Basis of Mitochondrial Genome Analysis
Source: PLoS One. 2016 Mar 3;11(3):e0149341. doi: 10.1371/journal.pone.0149341 (PMC4777564; doi:10.1371/journal.pone.0149341)

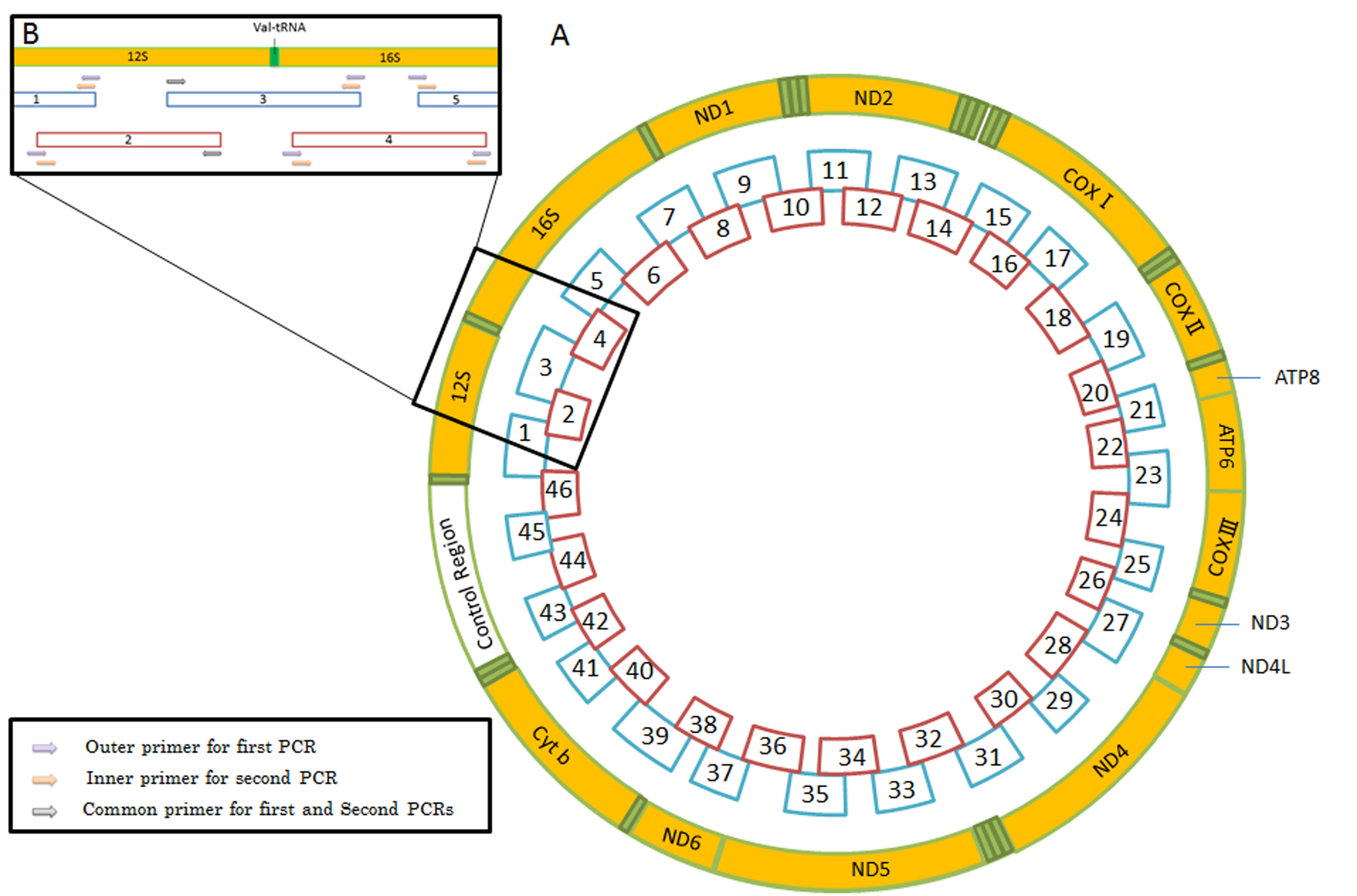

Supplement: S1 Fig — (A) Map of the otter mtGenome. (B) Positions of the outer primers, inner primers, and common primers. Circular genome (orange) showing the positions of two rRNAs, 13 proteins, 22 tRNAs (green boxes), and CR. Red (even number) and blue (odd number) frames indicate Set 1 and Set 2 of MPCR amplicons, respectively. (TIF) [file pone.0149341.s001.tif]

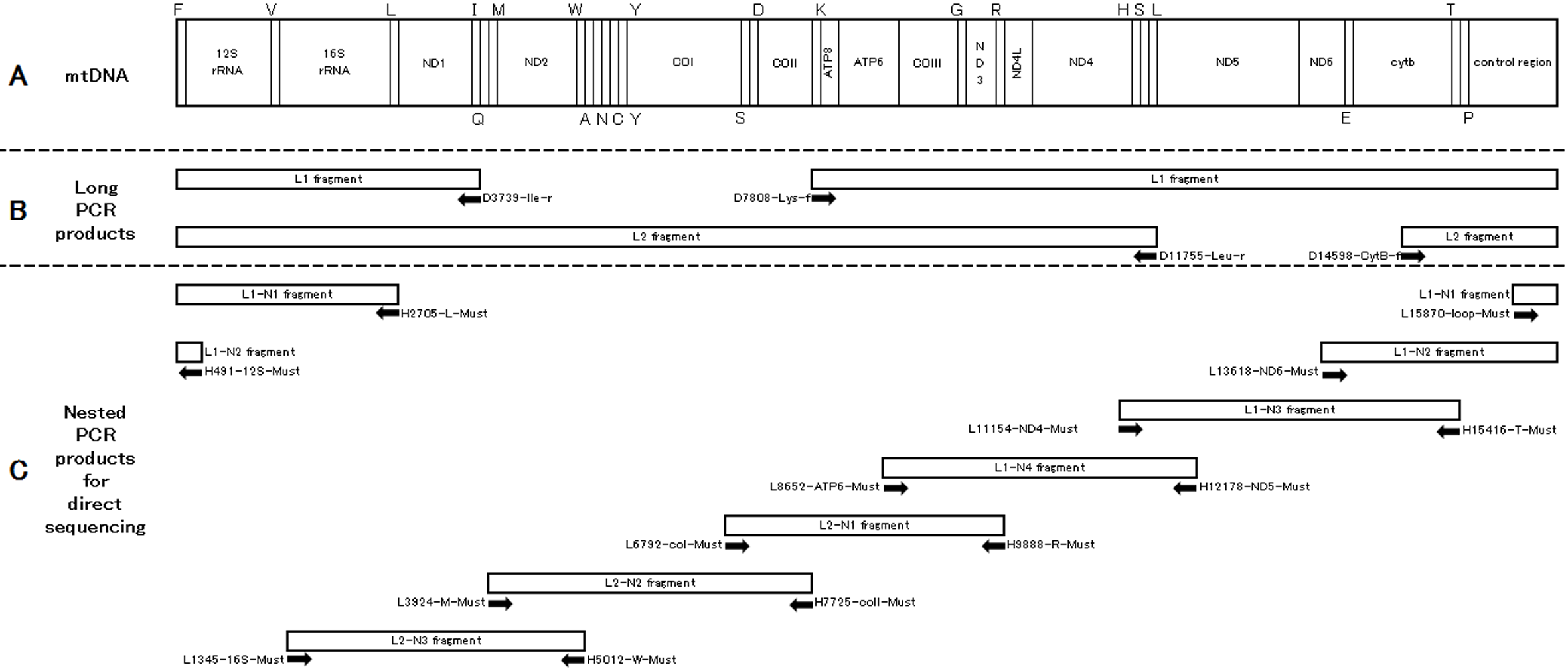

Supplement: S2 Fig — (A) 12 protein-coding genes are encoded by the H strand, but only the ND6 gene is encoded by the L strand. The 22 transfer RNAs are designated by single-letter amino acid codes. The RNAs encoded by the H strand and L strand are shown above and below the mtGenome maps, respectively. (B) Two segments (L1 and L2 fragments) that covered the mtGenome were amplified with two pairs of long range PCR primers. (C) To obtain templates for direct sequencing, nested PCR was performed using the long range PCR products as templates. Seven pairs of nested PCR primers amplified seven fragments (L1-N1, L1-N2, L1-N3, L1-N4, L2-N1, L2-N2, and L2-N3) that covered the mtGenome. (TIF) [file pone.0149341.s002.tif]

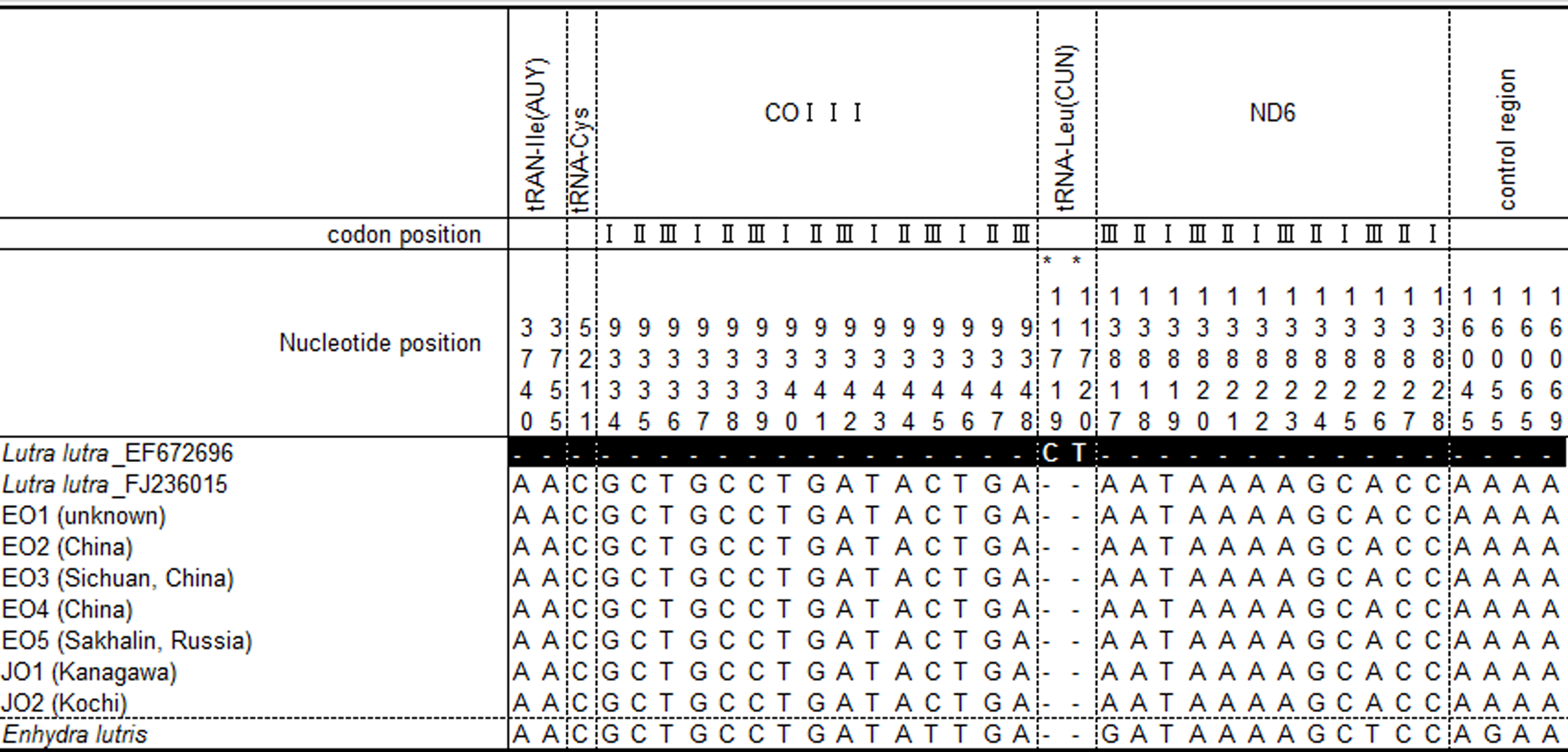

Supplement: S3 Fig — Dashes indicate gaps in the sequence alignment. E. lutris (sea otter) [34] is shown at the bottom of the alignment for comparison. The numbers of the nucleotide positions are based on the nucleotide positions in the Eurasian otter (GenBank accession No. LC049377), except for the numbers marked by asterisks. The numbers marked by asterisks are based on the nucleotide positions defined in the South Korean Eurasian otter EF672696 [39]. (TIF) [file pone.0149341.s003.tif]

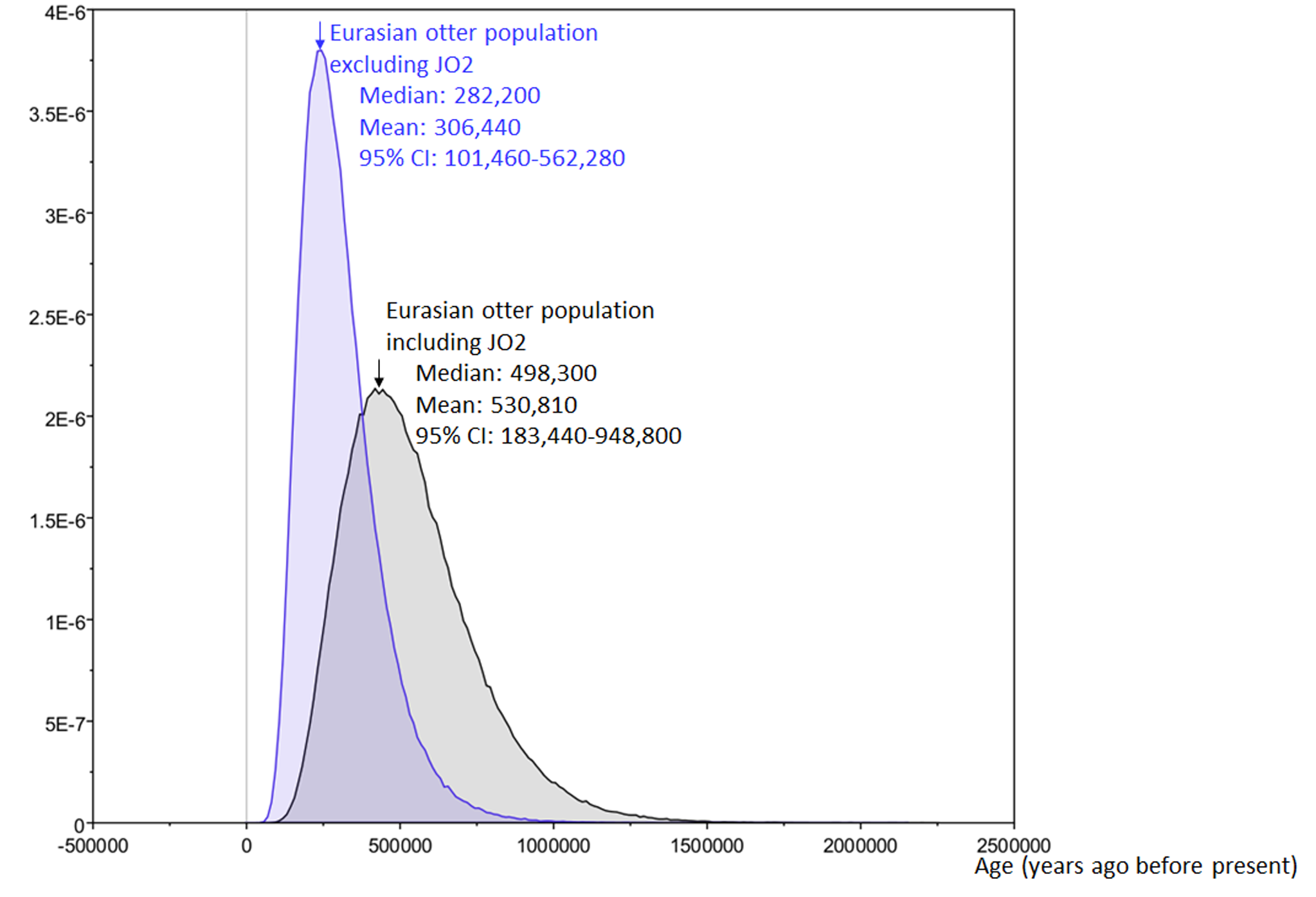

Supplement: S4 Fig — The posterior probability distribution of the tMRCA was inferred from the mitochondrial control regions using the coalescent method with the BEAST program. The vertical axis indicates the posterior probabilities and the horizontal axis indicates the tMRCA (as years before present). The posterior probability distribution colored in blue denotes the population of Eurasian otters excluding JO2, and the distribution colored in black denotes the population including JO2. (TIF) [file pone.0149341.s004.tif]

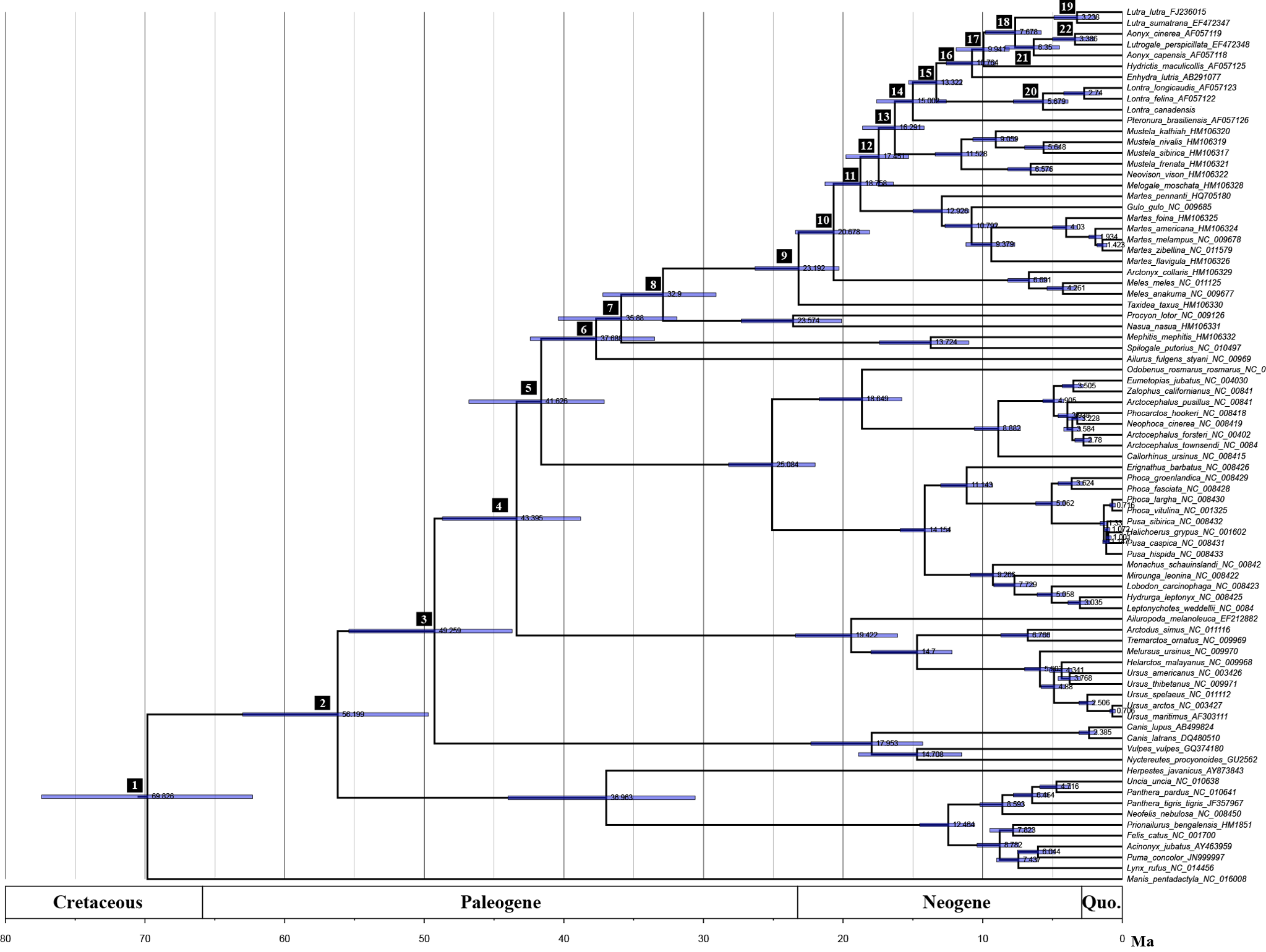

Supplement: S5 Fig — The nodal number indicates the estimated divergence time. Horizontal dark gray bars show the 95% credibility interval for the divergence time. Numbered boxes denote nodes. (TIF) [file pone.0149341.s005.tif]

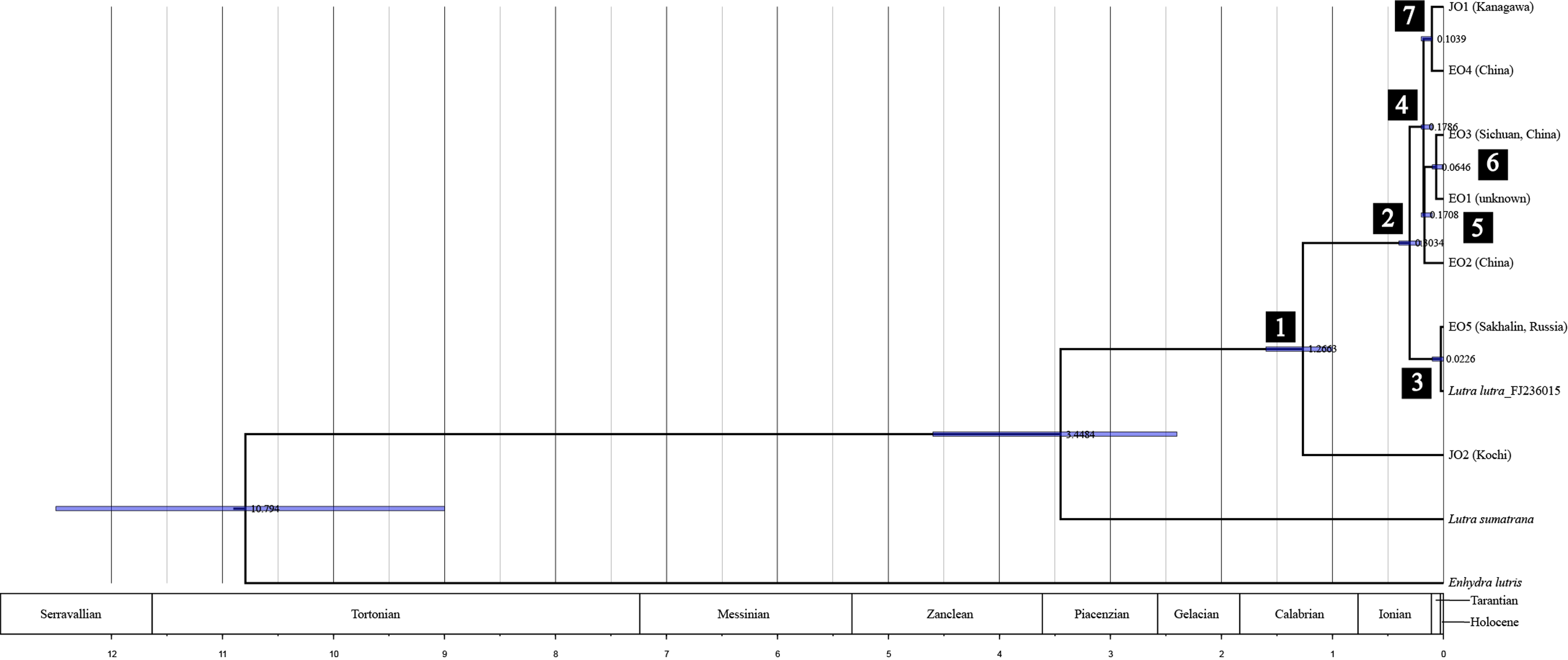

Supplement: S6 Fig — The nodal number indicates the estimated divergence time. Horizontal dark gray bars show the 95% credibility interval for the divergence time. Numbered boxes denote nodes. (TIF) [file pone.0149341.s006.tif]

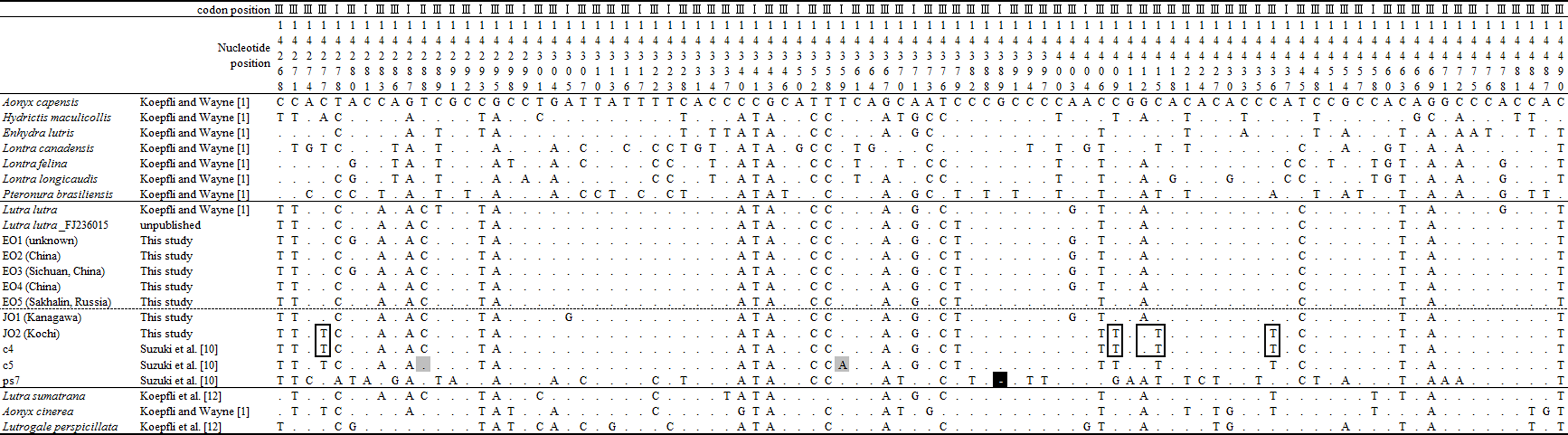

Supplement: S7 Fig — Nucleotide positions that are identical to those in the Aonyx capensis sequence are denoted with a period (.). Dashes indicate gaps in the sequence alignment. The numbers of the nucleotide positions are based on the nucleotide positions in the Eurasian otter (FJ236015). Letters highlighted in black indicate deletion site in the ps7. Letters highlighted in gray indicate the mutation sites between the c5 and the c4. Letters boxed by bold line indicate the mutation sites between seven individuals of L. lutra and the c4 + JO2. (TIF) [file pone.0149341.s007.tif]

**S3 File. Esri permission**


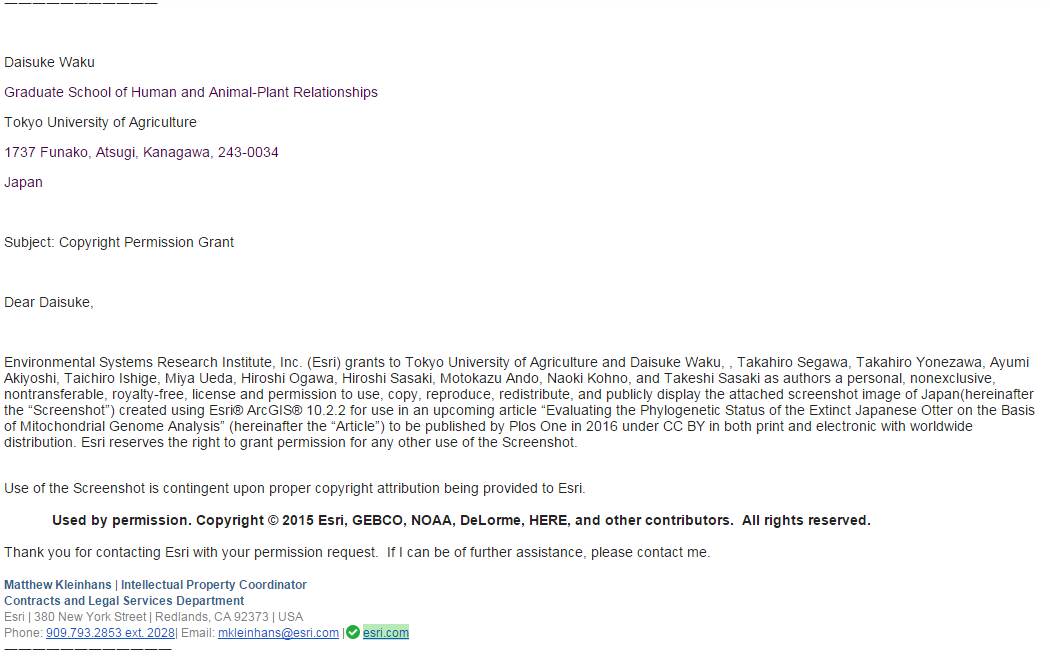

Supplement: S3 File — (DOCX) [file pone.0149341.s010.docx]
