# Supplementary material for: Evaluating the Phylogenetic Status of the Extinct Japanese Otter on the Basis of Mitochondrial Genome Analysis
Source: PLoS One. 2016 Mar 3;11(3):e0149341. doi: 10.1371/journal.pone.0149341 (PMC4777564; doi:10.1371/journal.pone.0149341)
Supplement: S1 File — (DOCX) [file pone.0149341.s008.docx]

**S1 File. Estimation of an ortholog of the *cytb* gene in *the* Japanese otter (Ehime)**

We identified the orthologous sequence of the *cytb* gene of the Japanese otter (Ehime) that was determined by Suzuki et al. [1]. Suzuki et al. [1] reported two types of *cytb* sequences (c4 and c5 clones) and one type of *cytb*-like sequence (ps7 clone) from the Japanese otter (Ehime). To estimate the orthologous sequence of the *cytb* gene from c4, c5, and ps7 sequences, we compared those sequences with *cytb* sequence data shown in Table 1 and S3 Table. The ps7 indicated a single nucleotide deletion at nucleotide position 14,389 (S7 Fig., highlighted in black). This deletion induced a frameshift mutation that produced an amino acid change from valine (GTA) to stop codon (TAG) at amino acid position 98. Therefore, we concluded that the ps7 was not an ortholog of the *cytb* gene in mitochondrial DNA. The c5 was nearly identical to the c4; however, the c5 differed from the c4 at two sites (S7 Fig., highlighted in gray). At nucleotide position 14,288, the c5 and the c4 indicated thymine and cytosine, respectively. The orthologous site of this position in *L.* *lutra*, *L.* *sumatrana*, JO1, and JO2 indicated cytosine. These substitutions altered the amino acid from threonine (ACC) to isoleucine (ATC) at amino acid position 39 in the c5. Additionally, at nucleotide position 14,359, the c5 indicated adenine, but other *cytb* sequences of Lutrinae indicated thymine. This nucleotide substitution altered the amino acid structure from phenylalanine (TTC) to isoleucine (ATC) at amino acid position 63 in the c5. We estimated that the c5 is not an orthologous sequence of the *cytb* gene. On the other hand, we could not observe nucleotide mutation between the c4 and JO2. The c4 and JO2 differed from eight sequences of *L.* *lutra* at five sites (S7 Fig., boxed by a bold line); however, those substitutions did not produce an amino acid substitution. From the above observation, we estimated the c4 as orthologous sequence of *cytb* gene in mitochondrial DNA of the Japanese otter (Ehime).

**Reference**

1. Suzuki T, Yuasa H, Machida Y. Phylogenetic position of the Japanese river otter *Lutra nippon* inferred from the nucleotide sequence of 224bp of the mitochondrial cytochrome *b* gene. Zool Sci. 1996;13(4): 621-626. doi: http://dx.doi.org/10.2108/zsj.13.621
