# Supplementary material for: Evaluating the Phylogenetic Status of the Extinct Japanese Otter on the Basis of Mitochondrial Genome Analysis
Source: PLoS One. 2016 Mar 3;11(3):e0149341. doi: 10.1371/journal.pone.0149341 (PMC4777564; doi:10.1371/journal.pone.0149341)
Supplement: S2 File — (DOCX) [file pone.0149341.s009.docx]

**S2 File. Coalescent analysis of Eurasian otters on the basis of mitochondrial DNA data**

The mitochondrial tMRCA was estimated by the coalescent method using BEAST ver. 1.7.4 [1]. For the European population, partial sequence data for the CRs were obtained from previous studies [2–4]. The homologous sites in the Asian population were also determined from the mtGenome data analyzed in the present study. The final dataset comprised 566 individuals, including the European population (557 individuals), Asian population (7 individuals), and Japanese population (2 individuals). The molecular clock could not be rejected; therefore, the estimated molecular substitution rate of 1.91 × 10^−8^/site/year in this region was employed to estimate the tMRCA while assuming a strict molecular clock. The HKY85+Γ model [5,6] was used as the nucleotide substitution model. A constant population size throughout time was assumed. The total length of MCMC was 500,000,000 generations. Trees were sampled every 1,000 generations. The first 50,000,000 generations were discarded as a burn-in. The convergence of the parameters was confirmed using the TRACER ver. 1.5 program by checking that all of the effective sample sizes exceeded 200.

**Reference**

1. Drummond AJ, Rambaut A. BEAST: Bayesian evolutionary analysis by sampling trees. BMC Evol Biol. 2007;7: 214. doi:10.1186/1471-2148-7-214
2. Stanton DWG, Hobbs GI, Chadwick EA, Slater FM, Bruford MW. Mitochondrial genetic diversity and structure of the European otter (*Lutra lutra*) in Britain. Conserv Genet. 2009;10(3): 733-737. doi: 10.1007/s10592-008-9633-y
3. Finnegan LA, Neill LO. Mitochondrial DNA diversity of the Irish otter, *Lutra lutra*, population. Conserv Genet. 2010;11(4): 1573-1577. doi: 10.1007/s10592-009-9955-4
4. Honnen AC, Petersen B, Kaβler L, Elmeros M, Roos A, Sommer RS et al. Genetic structure of Eurasian otter (*Lutra lutra*, Carnivora: Mustelidae) populations from the western Baltic sea region and its implication for the recolonization of north-western Germany. J Zool Syst Evol Res. 2010;49(2): 169-175. doi: 10.1111/j.1439-0469.2010.00582.x
5. Hasegawa M, Kishino H, Yano T. Dating of the human-ape splitting by a molecular clock of mitochondrial DNA. J Mol Evol. 1985;22(2): 160-174.
6. Yang Z. Among-site rate variation and its impact on phylogenetic analyses. Trends Ecol Evol. 1996;11(9): 367-372. doi:10.1016/0169-5347(96)10041-0
